# The role of microglia in Neuroinflammation associated with cardiopulmonary bypass

**DOI:** 10.3389/fncel.2024.1496520

**Published:** 2024-12-17

**Authors:** Lingda Meng, Tianxiang Gu, Peng Yu, Zhiwei Zhang, Zhijing Wei

**Affiliations:** ^1^Department of Cardiac Surgery, The First Affiliated Hospital of China Medical University, Shenyang, China; ^2^Department of Trauma Center, The First Affiliated Hospital of China Medical University, Shenyang, China

**Keywords:** microglia, neuroinflammation, signaling pathways, cardiopulmonary bypass, deep hypothermic circulatory arrest

## Abstract

Cardiopulmonary bypass (CPB) and deep hypothermic circulatory arrest (DHCA) are indispensable core techniques in cardiac surgery. Numerous studies have shown that cardiopulmonary bypass and deep hypothermic circulatory arrest are associated with the occurrence of neuroinflammation, accompanied by the activation of microglia. Microglia, as macrophages in the central nervous system, play an irreplaceable role in neuroinflammation. Current research on neuroinflammation induced by microglia activation mainly focuses on neurodegenerative diseases such as Alzheimer’s disease, Parkinson’s disease, neuropathic pain, acquired brain injury, and others. However, there is relatively limited research on microglia and neuroinflammation under conditions of cardiopulmonary bypass and deep hypothermic circulatory arrest. The close relationship between cardiopulmonary bypass, deep hypothermic circulatory arrest, and cardiac surgery underscores the importance of identifying targets for intervening in neuroinflammation through microglia. This could greatly benefit cardiac surgery patients during cardiopulmonary bypass and the perioperative period, significantly improving patient prognosis. This review article provides the first comprehensive discussion on the signaling pathways associated with neuroinflammation triggered by microglia activation, the impact of cardiopulmonary bypass on microglia, as well as the current status and advancements in cardiopulmonary bypass animal models. It provides new insights and methods for the treatment of neuroinflammation related to cardiopulmonary bypass and deep hypothermic circulatory arrest, holding significant importance for clinical treatment by cardiac surgeons, management strategies by cardiopulmonary bypass physicians, and the development of neurologically related medications.

## 1 Introduction

Cardiopulmonary bypass (CPB) is a crucial technique in cardiac surgery. Since the invention of the artificial heart-lung machine by American physician Gibbon in 1953, cardiac surgery has continuously progressed. In the 1970s, Griepp and colleagues developed the deep hypothermic circulatory arrest (DHCA) technique, which provides brain protection for patients undergoing aortic arch surgery (Griepp et al., 1975). By suppressing cerebral metabolic levels, this technique minimizes ischemic brain damage, ensuring a safe circulatory arrest period, reducing intraoperative bleeding, and enhancing surgical visibility and maneuverability. CPB and DHCA techniques have significantly advanced in the realm of cardiac surgery, yet the associated organ damage remains a challenge to mitigate. The brain, being the most susceptible organ to ischemia, is subject to various types of damage during cardiopulmonary bypass and deep hypothermic circulatory arrest, such as mechanical injury, ischemic–hypoxic injury, reperfusion injury, and inflammatory damage. One prominent manifestation of these injuries in the central nervous system is neuroinflammation. When neuroinflammation levels are elevated, it can lead to nerve damage and neuronal death, resulting in a series of neurological complications including cognitive dysfunction, memory impairment, and behavioral abnormalities. Cardiopulmonary bypass, a technique that temporarily maintains blood circulation to various organs in the body, partially simulates physiological processes. However, factors inherent in cardiopulmonary bypass such as anesthesia, blood dilution, changes in coagulation function, alterations in pump flow, and direct blood contact with artificial materials are unavoidable triggers of neuroinflammation (Jufar et al., 2021; Habes et al., 2023; Durandy, 2014).

Numerous studies have shown that the activation of microglia is a key feature of neuroinflammation (Woodburn et al., 2021; Bhusal et al., 2023), with some research involving cardiopulmonary bypass and deep hypothermic circulatory arrest. Ludmila Korotcova and colleagues, using a porcine cardiopulmonary bypass model, observed prolonged activation of microglia, confirming acute and long-term cellular responses to brain white matter injury induced by cardiopulmonary bypass. Although this persistent inflammatory response does not manifest changes in systemic biomarkers, it remains relatively silent, yet brain damage is indeed present. Activated and proliferating microglia exhibit neurotoxicity, affecting adjacent neurons and glial cells, thus exacerbating the damage (Korotcova et al., 2015). Khalid Elsaafien and collaborators, utilizing a sheep model, demonstrated that the increase in inflammatory factors triggered by cardiopulmonary bypass far exceeds that caused by general anesthesia and conventional cardiac surgical procedures like sternotomy; even in the absence of ischemia-hypoxia, cardiopulmonary bypass-related inflammatory reactions persist (Elsaafien et al., 2023). Ting Liu et al. using a rat cardiopulmonary bypass model, provided evidence from the perspective of postoperative cognitive dysfunction of the damaging effects of cardiopulmonary bypass. Following cardiopulmonary bypass, hypoxia in the hippocampal region induced an inflammatory response, activating microglia to produce more pro-inflammatory factors, creating a vicious cycle. Concurrently, hippocampal hypoxia resulted in blood–brain barrier damage, collectively leading to postoperative cognitive dysfunction in the nervous system (Liu et al., 2022). Katherine Giuliano and colleagues utilized a canine cardiopulmonary bypass model to demonstrate that the addition of hypothermic circulatory arrest to cardiopulmonary bypass leads to an increase in neural damage and inflammatory gene expression compared to conventional cardiopulmonary bypass alone. Dogs subjected to 2 h of hypothermic circulatory arrest exhibited sustained brain injury and behavioral deficits, accompanied by widespread activation of microglia, with the hippocampal tissue being most severely affected (Giuliano et al., 2021). Various animal models have confirmed the neurologic damage caused by cardiopulmonary bypass and deep hypothermic circulatory arrest, with a close association to microglia. The morphological changes in microglia serve as direct pathological evidence of neuroinflammation, highlighting the significant clinical implications of investigating microglia during cardiopulmonary bypass and deep hypothermic circulatory arrest. Therefore, it is reasonable to speculate that microglia play a crucial role in these processes and their function may involve intervention and modulation of neuroinflammation.

**Figure 1:**
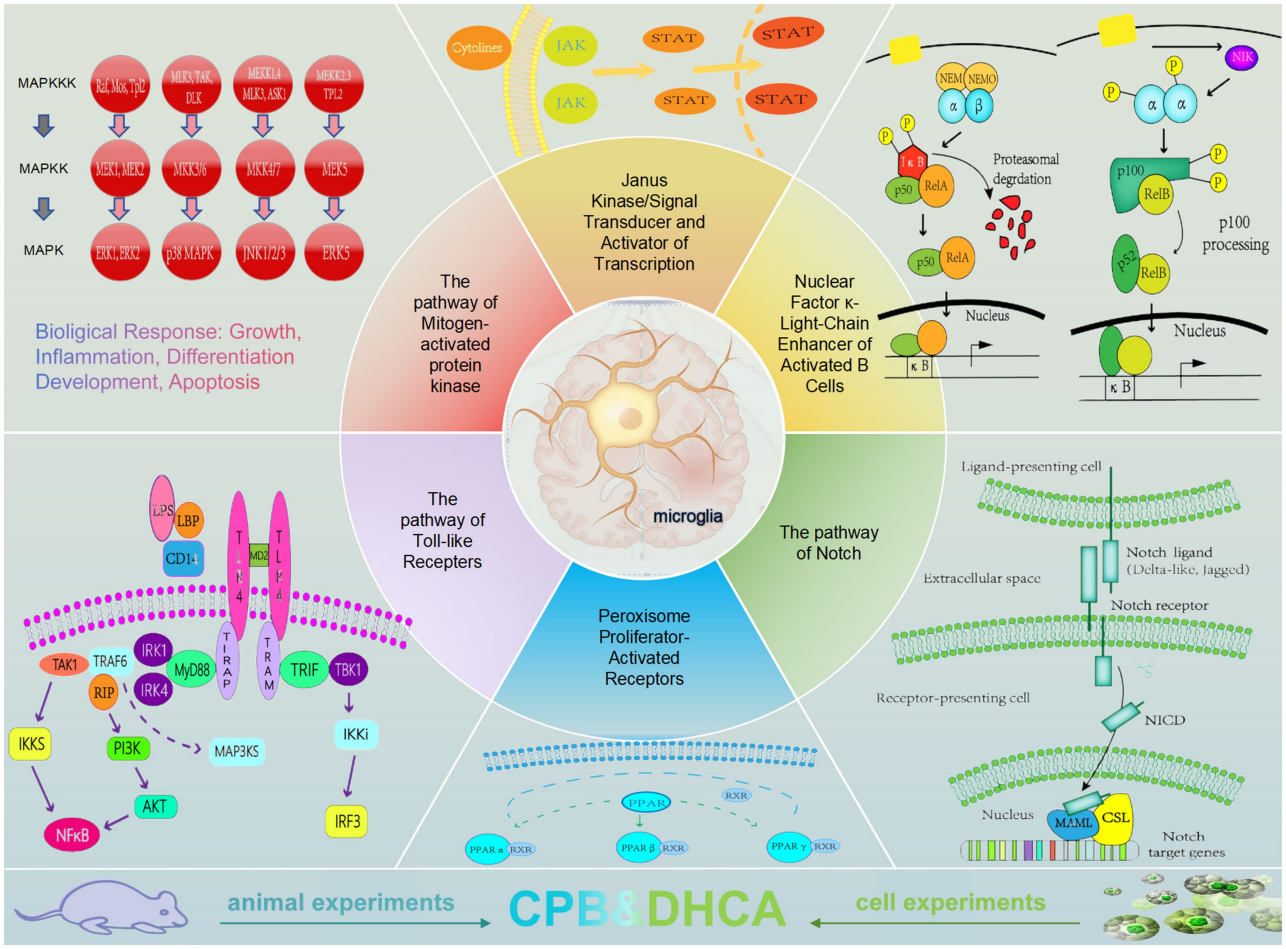
The signaling pathways of microglia in neuroinflammation.

The current research focus on neuroinflammation triggered by microglial activation primarily centers around neurodegenerative diseases such as Alzheimer’s and Parkinson’s, neuropathic pain, and acquired brain injury, among others. There is relatively limited research on microglia under conditions of cardiopulmonary bypass and deep hypothermic circulatory arrest, which are closely related to cardiac surgery. Currently, patients undergoing cardiac surgery under cardiopulmonary bypass still have a relatively high incidence of postoperative neurological complications, especially in cases involving the arch of the aorta requiring deep hypothermic circulatory arrest for total arch replacement, such as in patients with type A aortic dissection or aortic arch aneurysm. These patients may exhibit various postoperative manifestations, including delayed emergence from anesthesia, delirium, agitation, depression, cognitive impairment, and more. Prolonged activation of microglia and neuroinflammation are significant factors contributing to brain injury related to cardiopulmonary bypass and deep hypothermic circulatory arrest, leading to poor outcomes.Therefore, studying the function of microglia and the related molecular mechanisms under cardiopulmonary bypass and deep hypothermic circulatory arrest conditions holds significant scientific and clinical value. The present article provides a summary of the signaling pathways involved in neuroinflammation related to cardiopulmonary bypass and deep hypothermic circulatory arrest by microglia. It is the first analysis and summary of current interventions for neuroinflammation through cardiopulmonary bypass and deep hypothermic circulatory arrest, along with an examination of the research progress in relevant animal models. The advantages and limitations of large animal models and small animal models are discussed. Future research will continue to explore precise methods and targets for intervening with microglia to alleviate neuroinflammation associated with cardiopulmonary bypass and deep hypothermic circulatory arrest, ultimately achieving neuroprotection (Figure 1).

## 2 The signaling pathways of neuroinflammation and their relationship with microglia

Microglia originate from primitive macrophages in the yolk sac of the embryonic region and are also known as macrophages in neural tissue. Widely distributed in the central nervous system, they possess the ability to self-renew and maintain a relatively stable population. Microglia can phagocytize apoptotic neurons, clear apoptotic cells, mediate immune responses, and participate in tissue repair and regeneration, serving as a critical line of defense against various harmful stimuli for the brain. Furthermore, microglia play a crucial role in promoting brain development and maintaining brain function homeostasis by participating in synaptogenesis, exhibiting plasticity, transforming into different phenotypes, and displaying functional differences (Wolf et al., 2017; Nayak et al., 2014; Kwon and Koh, 2020). Under physiological conditions, microglia are evenly distributed with small cell bodies and highly branched morphology. Resting microglia exhibit typical multi-branched features, which increase the surface area of microglia, extend into the surrounding environment, constantly extending and retracting to search for toxic proteins phagocytizing neurons and irreparable neurons, and sensing changes in the local microenvironment. This branched structure enables the formation of synaptic connections with adjacent neurons to reshape neural circuits, playing an essential role in maintaining neuron survival and microenvironment homeostasis. Microglia are highly sensitive and can transition to an activated state upon exposure to pathogens, stress, or other pathological stimuli. Activated microglia exhibit enlarged cell bodies, retracted branches, migration, phagocytosis, and release of numerous cytokines and inflammatory mediators, leading to damage to glial cells and neurons. Traditional classifications include the classical activation type and the alternative activation type (Kettenmann et al., 2011; Umpierre and Wu, 2021; Prinz et al., 2019; Tang and Le, 2016). Classic activated microglia play a role in promoting inflammation, which can be induced by pro-inflammatory cytokines (INF-*γ*, TNF-*α*) and bacterial-derived stimuli (Liu et al., 2020). The activated microglia exhibit functions including inflammation inhibition, tissue repair, and immune regulation, contributing to neuroprotection. However, the traditional M1/M2 binary classification fails to demonstrate the full biological functions of microglia. Subsequently, researchers have defined resting, activated, ramified, ameboid, anti-inflammatory, and pro-inflammatory states. In this article, we use microglia activation to describe the process of disrupted microglia homeostasis transitioning from a physiological state to a pro-inflammatory state (Paolicelli et al., 2022; Marschallinger et al., 2020). The known signaling pathways are as follows:

### 2.1 The pathway of mitogen-activated protein kinase

**Figure 2:**
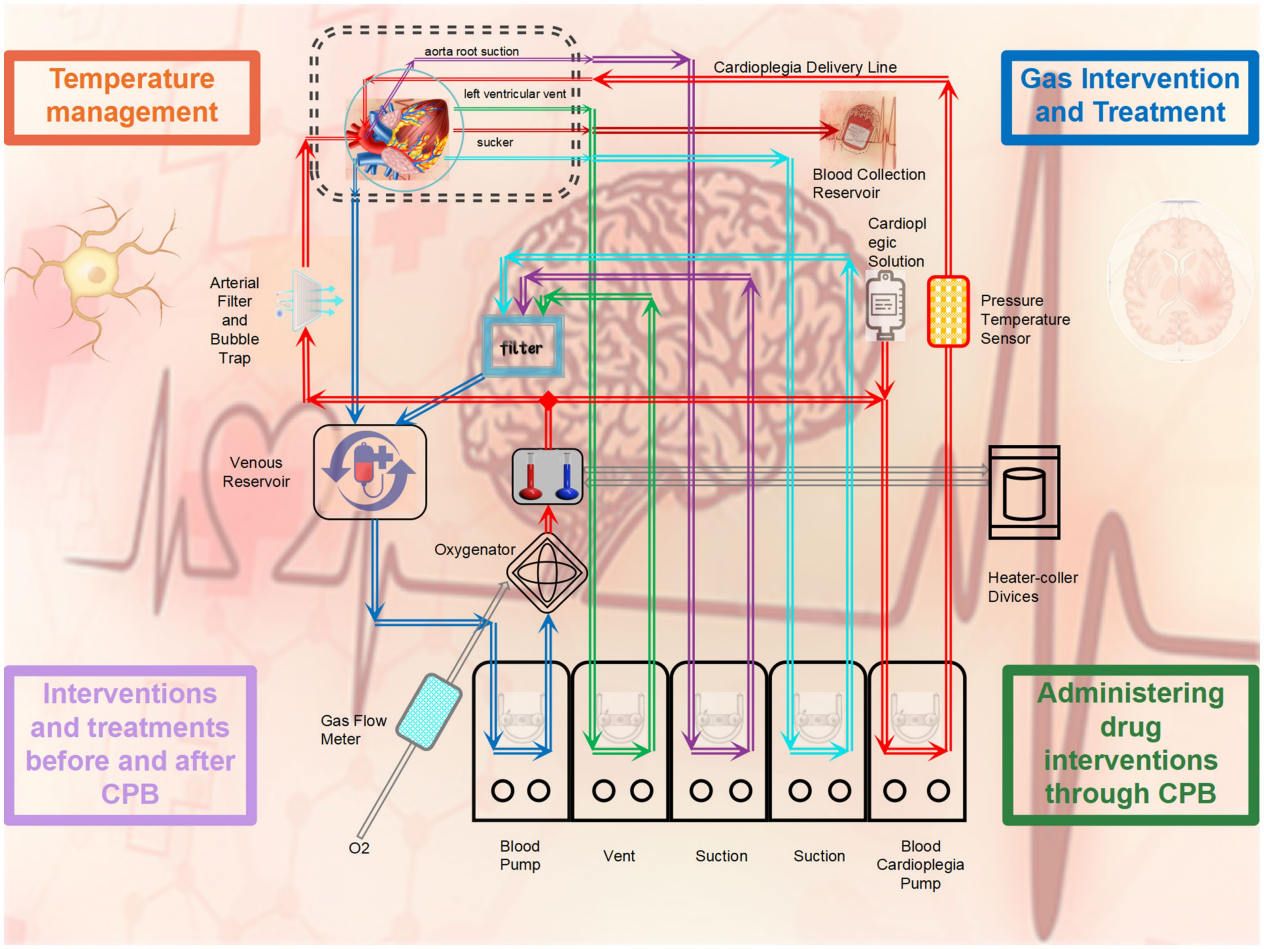
Intervention methods for microglia during cardiopulmonary bypass.

MAPK MAPK belongs to the highly conserved serine/threonine protein kinase family, comprising extracellular signal-regulated kinase (ERK), c-Jun N-terminal kinase (JNK), stress-activated protein kinase, p38, and MARK. It is a highly conserved signaling pathway in eukaryotic cells, where signals are transmitted from the cell membrane to the nucleus to exert their effects. The MAPK signal is activated through a three-tiered enzyme-catalyzed cascade reaction, where upstream proteins and specific receptors binding lead to the activation of MAPK kinase kinase (MAPKKK) and MAPK kinase (MAPKK) in sequence, resulting in the activation of MAPK. This pathway plays a crucial role in cellular processes such as proliferation, differentiation, apoptosis, and stress response (Figure 2).

#### 2.1.1 The pathway of extracellular signal-regulated kinase

ERK, short for Extracellular Signal-Regulated Kinase, is a signal transduction protein that transmits mitogen signals from cell surface receptors to the nucleus. ERK1 and ERK2 are the most well-known components of this pathway. The ERK pathway plays a crucial role in regulating cell proliferation, differentiation, and survival, serving as downstream effectors of various growth factors (Lu and Malemud, 2019). The ERK signaling pathway consists of Ras/Raf/MEK/ERK components. ERK1/2, positioned downstream of Ras, are essential signaling molecules in the Ras pathway, predominantly activated by growth factor receptors or protein tyrosine kinase receptors, requiring the involvement of Ras, PKC, and Raf proteins. In brain ischemia–reperfusion injury, the number of astrocytes and microglia cells, as well as the expression of ERK1/2 and calcium-dependent neutral protease-I 2, significantly increase. In this context, inhibiting ERK/calcium-dependent neutral protease-I2 signaling with PD98059 (an ERK inhibitor) or MDL28170 (a calcium protease inhibitor) substantially reduces the expression of these proteins, suggesting that blocking this signaling pathway can inhibit neuroinflammation and necrotic apoptosis associated with brain ischemia–reperfusion injury (Wang et al., 2021).

#### 2.1.2 The pathway of c-Jun N-terminal kinase

JNK, also known as c-Jun N-terminal kinase, is a crucial branch of the MAPK pathway that plays a significant role in various pathophysiological processes such as the cell cycle, cellular stress, reproduction, and apoptosis. It is primarily composed of JNK1, JNK2, and JNK3, with JNK1 and JNK2 being widely expressed throughout the body, while JNK3 is predominantly expressed in the brain, heart, and testes (Hagemann and Blank, 2001). The upstream kinases of JNK are MKK4 and MKK7, which activate JNK by dual phosphorylation at Thr183 and Thr185 sites. Upon activation by upstream signals, the nuclear transcription factor c-Jun is phosphorylated at Ser63 and Ser73 sites, enhancing its transcriptional activity. Phosphorylation of the amino-terminal end of c-Jun promotes the formation of c-Jun/c-Fos heterodimers and c-Jun homodimers, which bind to sites on the transcription activation protein-1 (AP-1), thereby increasing the transcriptional activity of certain genes (Garg et al., 2021). In BV-2 microglia, the specific JNK pathway inhibitor SP600125 can reduce JNK activity by downregulating phosphorylated inhibitory proteins, resulting in NF-κB inhibition. Moreover, in activated microglia, JNK activation precedes NF-κB activation (Cai et al., 2018).

#### 2.1.3 The pathway of p-38 mitogen-activated protein kinase

The p38 MAPK was discovered by Brewster in 1993 during the study of the impact of hyperosmotic environments on bacteria. It is a subclass of MAPKs, sharing similarities with JNK, and falls under stress-activated protein kinases (Brewster et al., 1993). The p38 MAPK pathway mediates stress responses such as inflammation and apoptosis, playing a significant role in apoptosis, cytokine production, transcription, and ischemia–reperfusion injury. The activation of the p38 MAPK pathway relies on a typical three-tier enzyme-catalyzed cascade reaction, with its cascade activity inducible by various stress factors and ligands, exerting its effects through different receptors including apoptosis-related receptors, GPCR receptors, and RTK receptors (Cuadrado and Nebreda, 2010). Activators of the p38 MAPK pathway are similar to those of the JNK pathway. Pro-inflammatory factors such as TNF-*α*, IL-1, and stress stimuli can also activate p38. Physical stress and osmotic shock are among the strong stimuli activating the p38 MAPK cascade reaction (Nagata et al., 1997). Numerous studies have confirmed the impact of the p38 MAPK pathway on microglia, for instance, Omega-3 docosapentaenoic acid polarizes microglia from M1 to M2 type, inhibits NF-κB and MAPK p38 signaling pathways activation, thereby protecting neurons from neuroinflammation (Liu et al., 2021). Acteoside B can activate NRF2 (Nuclear factor erythroid 2-related factor 2), inhibit the p38-MAPK signaling pathway, suppress glial scar formation, reduce tissue loss, alleviate demyelination, thus mitigating neuroinflammation and secondary neuronal apoptosis after spinal cord injury in mice (Xia et al., 2022).

#### 2.1.4 The pathway of extracellular signal-regulated kinase 5

ERK5, a distinct MAPK, plays crucial roles in stress and mitosis-induced cellular processes (Finegan et al., 2009). Upon stimulation, ERK5 phosphorylates various substrates, including transcription factors such as c-Myc, members of the MEF2 family, c-Fos, and SAP1a. Moreover, ERK5 can directly impact transcription through protein–protein interactions in its C-terminal non-catalytic region (Obara and Nakahata, 2010). Acting downstream of multiple innate immune receptors, ERK5 exhibits pro-inflammatory effects, with these receptors being associated with exogenous and endogenous inflammatory mediators (Wilhelmsen et al., 2015). Under high glucose conditions, exacerbation of neuroinflammation occurs through the inhibition of the MEK5/ERK5 signaling pathway, which appears to be a critical regulator of inflammatory responses and systemic glucose metabolism (Zhang et al., 2021). Furthermore, ERK5 is involved in post-inflammatory pain, as blocking the ERK5 signaling pathway in sensory neurons can prevent pain following inflammation (Katsura et al., 2007).

### 2.2 The pathway of Janus kinase/signal transducer and activator of transcription

The JAK/STAT signaling pathway serves as a fundamental conduit for the transmission of growth factors and cytokines involved in a variety of biochemical processes such as axon regeneration, inflammation, cell differentiation, proliferation, and death, making it a central node of cellular function (Oliva Jr et al., 2012). Mediating biological processes including hematopoiesis, immune adaptation, tissue repair, inflammation, adipogenesis, and cell apoptosis, the evolutionarily conserved JAK/STAT pathway consists of ligand-receptor complexes, JAK, and STAT components (Nabavi et al., 2019). The JAK family, comprised of non-receptor tyrosine protein kinases, includes four members: JAK1, JAK2, JAK3, and TYK2 (Jain et al., 2021). In the classical JAK/STAT pathway, ligand-receptor binding induces JAK phosphorylation, leading to STAT phosphorylation and subsequent formation of homodimers or heterodimers through SH2 domain-tyrosine phosphorylation interactions (Liu et al., 2023). Chen et al. observed upregulation of Enhancer of Zeste Homolog 2 (EZH2) in microglia under conditions of *in vivo* ischemia–reperfusion injury and *in vitro* glucose oxygen deprivation. Post ischemia–reperfusion injury, EZH2 promotes activation of pro-inflammatory microglia and downstream inflammatory responses. By inhibiting EZH2, DZNep disrupts STAT3 phosphorylation, mitigating inflammation associated with pro-inflammatory microglia and improving behavioral outcomes in ischemic stroke, reducing infarct volume, and exerting neuroprotective effects (Chen et al., 2019).

### 2.3 The pathway of nuclear factor *κ*-light-chain enhancer of activated B cells

NF-кB, initially discovered in 1986, is a protein complex that serves as a crucial nuclear transcription factor within cells, found in almost all animal cell types. It plays a vital role in various physiological processes such as inflammatory responses, immune reactions, cell apoptosis, and stress responses. The mammalian NF-кB family comprises five members: RelA/p65, c-Rel, RelB, p50 (NF-кB1), and p52 (NF-кB2). The canonical activation form of NF-кB involves the formation of a heterodimer composed of p50 or p52 subunits and p65. The activation of the NF-кB signaling pathway involves both canonical and non-canonical pathways (Gasparini and Feldmann, 2012). The canonical pathway is rapidly activated within minutes after exposure to pro-inflammatory signals, while the non-canonical pathway requires specific receptor activation (Hoffmann and Baltimore, 2006). The canonical NF-кB pathway can be activated by stimuli such as TNK-*α*, LPS, IL-1β, through cell surface receptors like IL-1R, TLR, TNFR, and antigen receptors, mediated by various adaptor proteins and the signaling kinase IKK complex. The non-canonical activation of NF-кB occurs through the induction of TNFR family ligands, leading to the activation of downstream receptors, promoting the activation of NF-кB-induced NF-kB-inducing kinase, subsequent phosphorylation of protein kinase IKKa, phosphorylation and degradation of NF-кBp100, resulting in the formation of NF-eB p52/RelB heterodimers, further phosphorylation and degradation of NF-кBp105 to form NF-кBp50/RelB heterodimers that regulate the transcription of target genes in the nucleus (Derudder et al., 2003). Studies have indicated an increase in cold-inducible binding protein in microglia in DHCA rat models, promoting the release of pro-inflammatory cytokines, potentially exacerbating neuroinflammation and neuronal damage through NF-кB signaling mediation (Liu et al., 2020). Adiponectin receptor agonists inhibit DHCA-induced acute neuroinflammation by promoting AMPK phosphorylation and suppressing the expression of proteins in the NF-кB pathway, inhibiting the activation of microglia in the rat hippocampus (Yan et al., 2022). Additionally, treatment of BV2 microglia with *Ginkgo biloba* leaf extract EGb761 inhibits the expression of inflammatory factors such as IL-6 and TNF-*α* under lipopolysaccharide stimulation, with involvement of the MAPK and NF-κB pathways in this process (Sun et al., 2024).

### 2.4 The pathway of notch

The Notch pathway is highly conserved during evolution, widely present in multiple species of invertebrates and vertebrates, participating in the development of almost all organs and helping to regulate tissue homeostasis post-organogenesis (Qian et al., 2019; Bray, 2016). It is closely associated with cell differentiation, proliferation, apoptosis, and endothelial-to-mesenchymal transition. Activation of the canonical Notch signaling pathway involves a three-step enzymatic cleavage process, where the cell surface Notch receptor becomes inactive upon ligand binding, leading to proteolytic cleavage by induced proteases. This results in the release of the intracellular Notch domain into the nucleus, where it binds with the transcriptional repressor RBP-Jκ to activate the transcription of target genes, thereby regulating cell proliferation, differentiation, and apoptosis (Zhou et al., 2022; Bray, 2006). Resveratrol inhibits the Notch signaling pathway, reducing spinal cord injury and exhibiting neuroprotective effects (Zhang et al., 2019). Lipoxin can modulate the phenotypic transformation of microglia following brain ischemia–reperfusion injury to exert neuroprotective effects, and inhibition of the Notch signaling pathway significantly alleviates the impact of lipoxin on microglial cell polarization (Li et al., 2021). Certain synthetic or naturally sourced drugs can influence neuroinflammatory responses, with celastrol regulating the activation of microglia through the Notch pathway, inducing morphological and functional changes in activated microglia (Yuan et al., 2015).

### 2.5 The pathway of peroxisome proliferator-activated receptors

PPARs, known as peroxisome proliferator-activated receptors, are members of the nuclear receptor family and function as ligand-activated transcription factors. This group comprises three subtypes: PPARα, PPARδ/*β*, and PPAR*γ* (Korbecki et al., 2019). The PPAR pathway primarily plays a role in lipid and glucose metabolism, inflammation, and cancer. PPARα is primarily involved in regulating fatty acid transport, esterification, and oxidation; PPARδ/β is associated with fatty acid oxidation and glucose uptake; while PPARγ regulates adipocyte differentiation, lipid storage, and insulin sensitivity (Kawada, 1998). Additionally, PPARs can negatively regulate gene expression without binding to DNA, directly inhibiting the activity of other transcription factors. In inflammatory responses, PPAR-γ exerts anti-inflammatory effects by competitively inhibiting inflammation signaling pathways and the production of inflammatory mediators. These inflammatory signaling pathways include JAK–STAT, NF-кB, activated T cell nuclear factor, and AP-1 (Chen et al., 2012).

### 2.6 The pathway of toll-like recepters

TLR, a transmembrane protein and a type of pattern recognition receptor (PPRs), is widely distributed in organs such as the heart, brain, lungs, liver, and kidneys, as well as in immune cells like dendritic cells and macrophages, mediating both innate and adaptive immunity (Dasari et al., 2011). Serving as the first line of defense against invading pathogens, TLR plays a crucial role in immune cell regulation, inflammation, cell survival, and proliferation. MyD88 serves as the main adaptor protein in the TLR signaling pathway, with its C-terminus binding to the TIR domains of TLRs, IL-1R, and IL-8R, and its N-terminus containing a death domain (DD). Upon recognition of specific PAMPs by the extracellular domain of TLRs, the TIR domain undergoes a conformational change, recruiting MyD88 or other adaptor proteins at the C-terminus. Subsequently, MyD88 recruits IL-1 receptor-associated kinase (IRAK) through the DD, followed by activation of downstream signaling through molecules such as tumor necrosis factor receptor-associated factor 6 (TRAF6), TGF-*β*-activated kinase 1 (TAK1), TAK binding proteins 1 and 2 (TAB1, TAB2), leading to the activation of nuclear factor-kappa B (NF-кB) or activator protein-1 (AP-1), inducing the expression of inflammatory cytokine genes like IL-1, IL-6, IL-8, IL-12, and TNF-*α* (Takeda and Akira, 2004; Luo et al., 2019). Studies suggest that melatonin inhibits inflammasome activation by downregulating TLR4/NF-кB pathway, reducing the expression of TLR4 receptors and its adaptor protein MyD88, thus decreasing inflammation (Hardeland, 2021). In a rat deep hypothermic circulatory arrest model, remifentanil downregulates the expression of upstream molecules in the TLR4 pathway, inhibiting neuroinflammation and reducing neuronal damage, an effect that can be reversed by lipopolysaccharide (Mao et al., 2024).

In general, the transition of microglia from a steady state to an activated state under cardiopulmonary bypass and deep hypothermic circulatory arrest involves numerous signaling pathways related to neuroinflammation, as evidenced by animal experiments and relevant cell studies. Animal experiments directly utilize models of cardiopulmonary bypass and deep hypothermic circulatory arrest in rats, while cell experiments employ conditions such as lipopolysaccharide (LPS) induction and oxygen–glucose deprivation (OGD) to effectively simulate the processes of neuroinflammation and ischemic damage, thus reasonably mimicking the experimental conditions of cardiopulmonary bypass and deep hypothermic circulatory arrest. The combination of animal and cell experiments complements each other, laying a solid foundation for subsequent human experiments. Both animal and cell models have limitations; for instance, interspecies differences exist between rats and humans in the neuroimmune system, and cell cultures are unable to replicate responses under physiological conditions and the complex *in vivo* biological environment, with cell experiment procedures being extremely intricate. Due to the unique nature of cardiopulmonary bypass as an experimental condition, it is not feasible to perform cardiopulmonary bypass procedures on humans or obtain human brain tissue, posing a significant challenge in this research field.

## 3 The modulation of microglia during cardiopulmonary bypass

During cardiopulmonary bypass, organ ischemia–reperfusion injury and contact activation of cardiopulmonary bypass itself are significant contributors to systemic inflammatory response. The systemic inflammation exacerbates neuroinflammation by intensifying the disruption of the blood–brain barrier. Within the central nervous system, microglia serve as the primary effectors of neuroinflammation. Under cardiopulmonary bypass conditions, the excessive activation of microglia leads to the release of various pro-inflammatory factors, further aggravating brain damage. Therefore, microglia represent a critical target for neuroprotective interventions during cardiopulmonary bypass and are considered a breakthrough in perioperative brain protection. In recent years, there has been a growing number of therapeutic interventions targeting microglia in the context of cardiopulmonary bypass, with various intervention methods showing promising results in animal models. These advancements lay a solid foundation for further in-depth research and offer new avenues for future clinical treatments.

### 3.1 The application of gases during cardiopulmonary bypass impacts the activation of microglia

The transformation of venous blood into arterial blood during cardiopulmonary bypass is a crucial step in the process. By utilizing an oxygenator for gas exchange, the oxygen concentration in the blood is increased, making the use of gases particularly important during cardiopulmonary bypass. Linardi et al. (2022) employed a deep hypothermic circulatory arrest rat model and, during circulatory arrest, directly connected gaseous NO to the oxygenator for antegrade brain perfusion. This method was found to reduce activation of microglia and neuroinflammation, decrease iNOS expression, and alleviate cell damage caused by inflammation activation (Linardi et al., 2022). Similarly, another study by Radovskiy et al. (2023) using a porcine cardiopulmonary bypass model demonstrated that adding nitric oxide to the oxygenator can inhibit the activation of microglia and neuronal degeneration after DHCA and ischemia–reperfusion, with the mechanism involving the MAPK–ERK pathway. By inhibiting the ERK signal, pro-inflammatory cytokines such as IL-6 are reduced, thereby exerting neuroprotective effects (Kajimoto et al., 2021). Kharnaf et al. (2024) utilized a rat cardiopulmonary bypass model and found that changing the oxygen gas supplied to the oxygenator from pure oxygen to 95% oxygen with 5% carbon dioxide did not lead to tissue hypoxia. Currently, gas interventions during cardiopulmonary bypass mainly involve exogenous hydrogen and nitric oxide, and their neuroprotective effects may be related to the reducing properties of these gases. Therefore, can noble gases such as inert gases exert neuroprotective effects? Many studies have already shown the protective effects of inert gases on traumatic brain injury, brain injury caused by cardiac arrest, and brain injury due to cerebral infarction (Terrando and Warner, 2019; Motta et al., 2024; Dandekar et al., 2022). Given that brain injury is inevitable during cardiopulmonary bypass, especially during deep hypothermic circulatory arrest, whether inert gases can influence neuroinflammation related to deep hypothermic circulatory arrest through microglia may be one of the future research directions.

### 3.2 One of the crucial factors affecting the function of microglia during cardiopulmonary bypass is the temperature maintained

Temperature management (TTM) during cardiopulmonary bypass is particularly crucial. To meet the needs of various cardiac surgeries, cardiopulmonary bypass requires cooling, rewarming, and sometimes circulatory arrest, making temperature regulation a focal point and challenge in cardiopulmonary bypass technology. Microglia play different roles under different temperature conditions during cardiopulmonary bypass. Stern et al. (2024) ound that in rats under different cardiopulmonary bypass temperatures (mild hypothermia and deep hypothermia), the neuroinflammatory response varied. Under mild hypothermic conditions, microglia were inhibited from M1 pro-inflammatory phenotype activation, while under deep hypothermic conditions, the activation level toward the M1 phenotype was significantly higher than that under mild hypothermia, with a stronger neuroinflammatory response observed in the amygdala and hippocampus (Stern et al., 2024). Data from Linardi et al. (2020) experimental research suggest that microglial cell activation can be used to assess inflammation cell activation in the brain. Compared to slow rewarming, rapid rewarming significantly increased microglial cell activation in the cerebral cortex and hippocampal tissues after low-temperature circulatory arrest, implying that slow rewarming following circulatory arrest may be associated with better neurological outcomes (Linardi et al., 2020). Another study also found similar evidence that delayed hypothermic therapy following deep hypothermic circulatory arrest can suppress microglial cell activation, reduce inflammation response, and protect brain tissue, with the protective effect and treatment duration of delayed hypothermic therapy being correlated (Muedra et al., 2022). Furthermore, in a rat model of cardiopulmonary bypass resuscitation after cardiac arrest, using the carotid artery as an additional route for arterial perfusion to selectively regulate cerebral perfusion and lower brain temperature rapidly at the start of resuscitation significantly reduced microglial cell activation and pro-inflammatory mediator levels, alleviating hippocampal tissue pathological damage. These studies underscore the importance of temperature management in cardiopulmonary bypass and deep hypothermic circulatory arrest (Zhai et al., 2022). The impact of microglia on neuroinflammation varies under different temperatures, especially during the rewarming phase where the duration of rewarming, perfusion strategies during rewarming, and other factors can influence the effectiveness of neuroprotection.

### 3.3 Treatment interventions before and after cardiopulmonary bypass surgery have shown significant efficacy with ample evidence

Appropriate pharmacological interventions and treatments before and after cardiopulmonary bypass surgery can alleviate neuroinflammation, improve cognitive function for brain protection through diverse intervention and treatment modalities. A study indicates that probiotics administered to rats regularly before cardiopulmonary bypass can alleviate the decline in learning and spatial memory abilities caused by the surgery, possibly by regulating inflammatory factors to reduce neuronal apoptosis in the hippocampal tissue (Zhang et al., 2024). Preconditioning with sevoflurane has been shown to mitigate brain damage induced by inflammation during cardiopulmonary bypass in rats, evidenced by reduced S-100β in serum and decreased neuronal apoptosis in hippocampal tissue, likely due to increased brain resilience to trauma, ischemia, and hypoxia (ZHOU et al., 2015). The use of a synthetic cannabinoid analog injected into the rat peritoneal cavity prior to deep hypothermic circulatory arrest can inhibit the activation of microglia, reduce inflammation levels, and alleviate hippocampal tissue damage post-arrest (Yu et al., 2023). Chlorogenic acid, a plant-derived compound with potent anti-inflammatory properties, when administered to rats following deep hypothermic circulatory arrest, can reduce levels of inflammatory cytokines, suppress relevant inflammatory pathways to prevent perioperative brain damage and cognitive complications (Chen et al., 2021). Immediate postoperative administration of erythropoietin B into the rat peritoneal cavity has been found to reverse the process of M1 polarization of microglia induced by cerebral ischemia, promote their transformation into the M2 phenotype, and alleviate ischemia-induced nerve damage (Chen et al., 2024). Additionally, post-cardiopulmonary bypass administration of minocycline to rats can improve neuroinflammation after cardiopulmonary bypass by reducing microglial cell activation and subsequently ameliorate postoperative cognitive dysfunction (Wang et al., 2022). Hence, pharmacological interventions and treatments during the peri-cardiopulmonary bypass period are crucial, particularly post-cardiopulmonary bypass when microglia are activated, where appropriate interventions significantly enhance the neuroprotective process.

### 3.4 Administering drug interventions through cardiopulmonary bypass is a feasible method with promising prospects

Numerous studies have confirmed that cardiopulmonary bypass is one of the effective routes for mesenchymal stem cell therapy, which is relatively safe and stable, serving as a unique therapeutic approach in cardiac surgery. Kei Kobayashi et al. compared the effects of different doses of mesenchymal stem cell therapy and found that both low and high doses of mesenchymal stem cell therapy shifted the pro-inflammatory phenotype of microglia to a low-activity state. Moreover, administering high doses of mesenchymal stem cell therapy has the potential to alleviate systemic inflammation and brain-specific inflammation caused by cardiopulmonary bypass. During rewarming, the delivery of mesenchymal stem cells via cardiopulmonary bypass altered the activation state of microglia from an activated phenotype to an inactivated phenotype (Kobayashi et al., 2023). Sarkislali et al. (2023) demonstrated in a young pig cardiopulmonary bypass model that intravenous infusion of bone marrow-derived mesenchymal stem cells can provide neuroprotection and reduce behavioral abnormalities induced by cardiopulmonary bypass. The experiments observed a sharp increase in the number of microglia induced by cardiopulmonary bypass, significant inhibition of excessive activation of STAT3, thereby reducing inflammation stress, microglial activation, and neuronal apoptosis during cardiopulmonary bypass (Sarkislali et al., 2023). Kamenshchikov et al. (2024) utilized a sheep model and found through comparative experiments that continuous delivery of nitric oxide into the extracorporeal pathway during cardiopulmonary bypass can alleviate the impact of extracorporeal circulation on red blood cell deformation. This intervention reduces red blood cell apoptosis and inflammation caused by endothelial damage, improves tissue hypoxia and inadequate perfusion, thereby exerting organ protective effects (Kamenshchikov et al., 2024).

## 4 The utilization and challenges of animal models in cardiopulmonary bypass

In In recent years, researchers have been dedicated to exploring cardiopulmonary bypass and deep hypothermic circulatory arrest animal models, which significantly contribute to simulating pathophysiological processes, enhancing perioperative management, and organ protection strategies during DHCA. In the process of establishing DHCA animal models, various animal species such as pigs, sheep, dogs, and rabbits have been experimented with (Fujii, 2020; Schnoering et al., 2013; Jungwirth and de Lange, 2010). Rats, being the preferred choice for small animal models of deep hypothermic circulatory arrest, offer numerous advantages. These include requiring less priming fluid for cardiopulmonary bypass to avoid blood dilution, having a cardiovascular anatomy similar to humans, exhibiting high homology between rat and human genes, being commercially available, showing minimal individual differences, providing rich means for gene and protein detection, offering relatively low cost, easy maintenance, and simple and accurate behavioral testing (Waterbury et al., 2011; Ballaux et al., 1999; Madrahimov et al., 2018). The challenge with rat DHCA lies in the survival and duration of survival post-DHCA. Researchers have been striving to overcome these challenges and have made significant progress. In recent years, researchers have progressively improved and optimized the rat model to make it more clinically relevant, allowing the rat DHCA model to assist researchers in obtaining various tissues and organs more effectively (Yan and Ji, 2022; Linardi et al., 2024). Despite the relative maturity and promising prospects of the rat cardiopulmonary bypass animal model, many challenges persist, such as the inability to strictly maintain aseptic conditions, mismatches in cardiopulmonary bypass circuits and priming fluids, difficulties in stopping cardiopulmonary bypass, vascular injuries from intubation, postoperative heart and respiratory failure, as well as challenges in postoperative suctioning, all of which can lead to shortened survival time or death in rats post cardiopulmonary bypass withdrawal.

The selection of large animals as cardiopulmonary bypass animal models is meaningful, as large animal models complement small animal models in certain aspects. Compared to small animals, large animals are closer to humans in terms of structure, function, and response, which is crucial for the development of instruments, evaluation of drug effects, etc. (Edinger et al., 2020). Currently, pigs and sheep are good choices for large animal cardiopulmonary bypass models. The similarities in volume, structure, and function between pig brains and human brains, pig hearts and human hearts, as well as the structural resemblance of the pig hippocampus to that of humans, make pigs a more scientifically accurate animal model for extracorporeal brain protection. Similarly, sheep as cardiopulmonary bypass models have similar advantages. For example, sheep are more docile animals suitable for long-term experiments, the placement and intubation methods of cardiopulmonary bypass devices in sheep are similar to those in humans, and the relevant monitoring equipment can be the same as for humans, making tissue sample collection easy and abundant. Khalid Elsaafien et al. conducted research using a sheep cardiopulmonary bypass model and found that in sheep, microglia in the frontal cortex exhibit an anti-inflammatory phenotype, while microglia in the parietal and temporal cortex exhibit a pro-inflammatory phenotype. During CPB, the additional production of pro-inflammatory cytokines exceeds the increase in pro-inflammatory factors caused by general anesthesia and thoracotomy, with CPB leading to widespread activation of small glial cells in the cerebral cortex (Elsaafien et al., 2023). However, large animal models also have some drawbacks, such as high cost, large space requirements, lack of good evaluation methods for cognitive function in the nervous system, and difficulty in control. Therefore, it is necessary to comprehensively consider various factors and select an appropriate animal model based on the experimental purposes and conditions.

## 5 Summary and outlook

The The cardiopulmonary bypass and deep hypothermic circulatory arrest models have been aiding researchers in exploring various mechanisms and addressing fundamental as well as clinical issues. In recent years, the exploration of relevant animal models has significantly assisted in simulating pathophysiological processes, enhancing perioperative management, and developing organ protection strategies during DHCA. Despite encountering various challenges in exploring animal models of cardiopulmonary bypass and deep hypothermic circulatory arrest, overall, animal models such as rats, pigs, and sheep have gradually matured and can effectively simulate human physiological processes. Future research should build upon existing findings and delve deeper into genomics, transcriptomics, proteomics, metabolomics, etc., to further investigate various pathways related to astrocyte activation induced by cardiopulmonary bypass, enabling precise interventions to reduce adverse reactions and complications. Moreover, preconditioning training is a breakthrough in cardiopulmonary bypass neuroprotection. Most cardiac surgeries are elective, allowing ample time for preconditioning during preoperative evaluation, such as pre-injection of relevant drugs and targeted exercises. From the perspective of cardiac surgery, intravenous administration of drugs during surgery through cardiopulmonary bypass is the most direct and rapid way, with promising applications. Researchers should continue efforts to explore relevant drugs, identify targets related to microglia activation or inhibition, reduce neuroinflammation during cardiopulmonary bypass, achieve neuroprotection, and thereby provide new insights for therapeutic interventions for cardiac surgeons during and after surgery.
